# Themes and variations: An exploratory international investigation into resuscitation decision-making^☆^

**DOI:** 10.1016/j.resuscitation.2016.01.020

**Published:** 2016-06

**Authors:** Alexander J.O. Gibbs, Alexandra C. Malyon, Zoë B. McC Fritz

**Affiliations:** a Department of Acute Medicine, Cambridge University Hospitals, Box 148, Hills Road, Cambridge, UK; b Warwick Medical School, University of Warwick Coventry, CV4 7AL, UK; c Gonville & Caius College, University of Cambridge, Cambridge, UK

**Keywords:** Resuscitation orders, International perspectives, Physician–patient relationship, Resuscitation decisions

## Abstract

**Background:**

Do Not Attempt Cardiopulmonary Resuscitation (DNACPR) decisions are made in hospitals throughout the globe. International variation in clinicians' perception of DNACPR decision-making and implementation and the factors influencing such variation has not previously been explored.

**Methods:**

A questionnaire asking how DNACPR decisions are made, communicated and perceived in their country was composed: it consisted of seven closed-answer and four open-answer questions. It was distributed to 143 medical professionals with prior published material relating to DNACPR decisions. Under-represented geographical areas were identified and an additional 34 physicians were contacted through medical colleagues and students at the university hospital from which this study was based. The respondents had 4 weeks to answer the questionnaire.

**Results:**

78 responses (44%) were received from 43 countries. All continents were represented. 88% of respondents reported a method for implementing DNACPR decisions, 90% of which discussed resuscitation wishes with the patient at least half of the time. 94% of respondents thought that national guidance for DNACPR order implementation should exist; 53% of countries surveyed reported existence of such guidance. Cultural attitudes towards death, medical education and culture, health economics and the societal role of family were commonly identified as factors influencing perception of DNACPR decisions.

**Conclusions:**

The majority of countries surveyed make some form of DNACPR decision but differing cultures and economic status contribute towards a heterogeneity of approaches to resuscitation decision-making. Adequacy of relevant medical education and national policy are two areas that were regularly identified as impacting upon the processes of DNACPR decision-making and implementation.

## Introduction

It has been recognised across geographical boundaries that certain medical interventions may cause more overall harm than benefit. One such intervention is attempted Cardiopulmonary Resuscitation (CPR).^1^, ^2^ Given that cardiopulmonary arrest is the ‘final common pathway’ for all of us, Do Not Attempt Cardiopulmonary Resuscitation (DNACPR) decisions have become common-place in many countries.^3^, ^4^

When contemplating a resuscitation decision, the four fundamental principles often applied in medical ethics should be considered: respect for patient autonomy; beneficence; non-maleficence; and justice with regards both to the allocation of finite resources, and to equal access to best available care without discrimination.^5^ The ethical principles which guide decision-making may vary depending upon the cultural, social and economic context in which the decision is made.^6^ Although studies on individual countries' practice^7^, ^8^ and comparing specific countries^9^ have been published, there is not, to our knowledge, a wider overview of DNACPR decisions.^10^ National websites publishing policy or guidance on resuscitation decisions are rare, and so international approaches are not easily synthesised. We have previously published on resuscitation decision-making variation across the UK.^8^ This study aims to examine international variation in clinicians' perception of DNACPR decisions and implementation and explores which factors influence such variation.

## Method

Physicians who had previously published material relating to DNACPR decisions (or similar end-of-life issues) within their country were identified via a ‘Pubmed’ search of do-not-resuscitate limited to the last 5 years. Papers not about DNACPR decisions were excluded, as were those from the UK, where the authors are based. Under-represented geographical areas were identified and additional respondents found through medical colleagues and students at the university hospital from which this study was based.

A questionnaire on local and national DNACPR policy, national attitudes towards DNACPR and other issues at the end-of-life was composed. Questions 1–7 were single answer questions; questions 8–11 were open-ended (Appendix A). The questionnaire and an invitation to participate in the study were sent to identified corresponding authors by email (Appendix B). It was estimated that the questionnaire required 10–15 min to complete. Respondents who did not have time to answer every question were encouraged to complete partially the questionnaire. A reminder email was sent after 2 weeks and recipients were given a total of 4 weeks to respond.

The free-text answers from questionnaires were independently analysed by two researchers (AG and AM) to identify themes which emerged from participant responses.

## Results

**Fig. 1:**
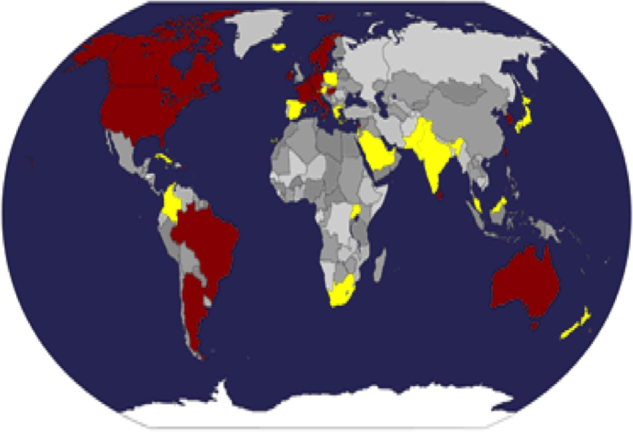
Map of the world:  – No national policy or guidance reported;  – National policy or guidance reported (when there was discrepancy between respondents from the same country, that country was placed in the “National policy or guidance reported” category).

**Fig. 2:**
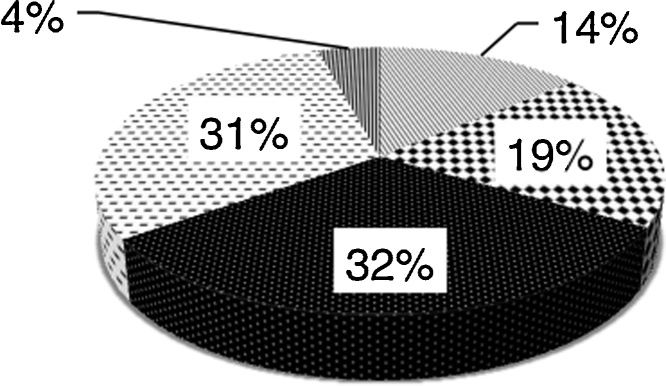
How often do you discuss decisions about resuscitation with patients and/or their family? 31% Always; 32% Most of the time; 19% Around half of the time; 14% Rarely; 0% Never; 4% No response.

**Fig. 3:**
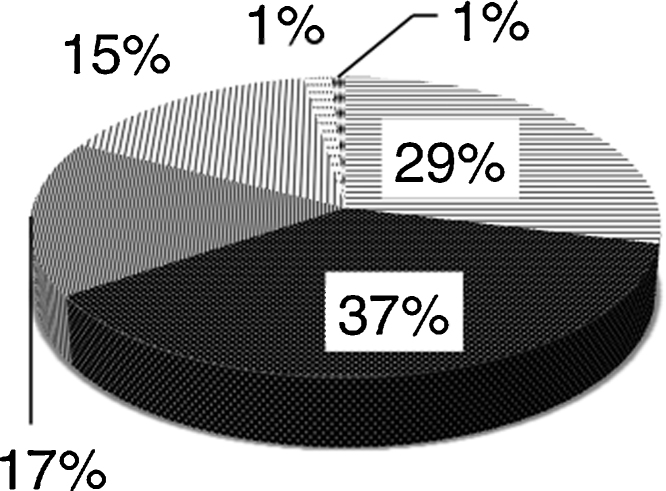
How do you communicate these decisions to other doctors in your institution? 29% Verbally; 37% Written in notes; 17% By completing a pre-printed document; 15% Electronically; 1% Other method; 1% No response.

**Fig. 4:**
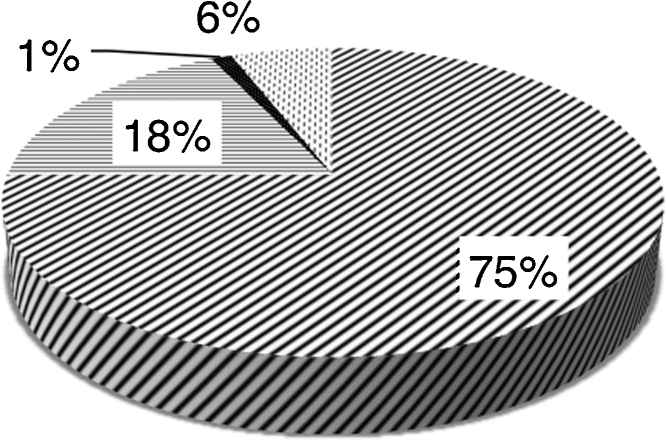
In what setting do most patients die within your country? 75% Hospital; 18% Home; 1% Nursing home; 0% Hospice; 6% No response.

The ‘PubMed’ search yielded 372 hits, 171 of which were about CPR decisions. 143 of these provided current email addresses. 58 authors on this list responded to the questionnaire. The majority of responses came from Europe, the Americas and Oceania. Purposive sampling from under-represented continents (Africa and Asia) identified a further 34 physicians, of whom 20 replied. In total 78 responses were received from 43 countries. Overall response rate was 44%. Fig. 1 displays the countries from which responses were received. 61 respondents completed every question from 1 to 8 (displayed in Fig. 2, Fig. 3, Fig. 4), whilst the remaining 17 provided partial responses. Full responses to questions 1–8 by country can be found in Appendix C.

### Making a DNACPR decision

88% of respondents reported a method for making DNACPR decisions: most had conversations at least ‘half of the time’. Respondents from Argentina, Hungary, Iceland, Ireland, Malaysia, Norway and Taiwan reported having conversations only ‘rarely’. Respondents from Brunei, Greece, Italy and South Africa reported working in hospitals without a method for making DNACPR decisions; those from Brunei and South Africa reported that they ‘rarely’ discuss CPR decisions with their patients.

58 of the 67 respondents (87%) working in a hospital where there was a method for making DNACPR decisions communicated these decisions to their medical colleagues in written format (‘in the notes’, ‘electronically’ or ‘by completing a pre-printed document’). Those from Argentina, Colombia, Cuba, Hungary, and one of the respondents from India, Japan, Norway, Saudi Arabia and Taiwan, said that decisions to withhold CPR were communicated ‘verbally’.

Respondents from 23 of the 43 countries sampled were aware of national guidance on DNACPR decision-making (Fig. 1). When asked whether they thought that there should be national policy/guidance for making resuscitation decisions, 94% of respondents (62/66, 12 not providing a response) said yes. Reasons for advocating for national policy or guidance included beliefs that it would: standardise decision-making; provide support for clinicians who may be concerned about medico-legal challenges; minimise the potential for conflict with patient's families; and legitimise clinical CPR decisions. One respondent from South Africa felt that ‘*clear guidelines*’ could ‘*make healthcare as equitable as possible*’. Respondents who did not support a national policy felt that the complexity of decisions relating to CPR would make it difficult to formulate effective guidance. One respondent from France wrote: ‘*Every patient is different. One can have broad directives but a national policy/guideline will not target individual patients and can produce a lot of disagreement.*’

### Place of death

73 respondents (from 42 nations) completed the question on place of death. Respondents from Cuba, Holland, India and Uganda said the majority died at home; respondents from Brazil, Malaysia, Poland and Singapore were divided as to whether the majority died at home or in hospital; Malta also included nursing homes; all other respondents reported that the majority died in hospital.

### Perceived influences on DNACPR decision-making

The final questions explored respondents' views on how DNACPR decisions and end-of-life care as a whole were perceived within their country. Responses were free-text.

Six main themes emerged. Table 1 presents associated quotes.

#### Societal and political factors

Cultural attitudes to death were mentioned by a number of respondents. Respondents from Japan, Singapore and Taiwan mentioned taboos surrounding discussing death, where it could be perceived as ‘*bad luck*’. A respondent from the USA described how ‘*a cultural aversion to death exists, with a perception that a DNACPR decision would be contrary to the ‘American ‘can-do’ attitude.*’ A South African respondent said that the low life expectancy in South Africa meant that ‘*most patients are accepting*’ of death. In the Netherlands, a ‘*relatively ‘open’ culture*’ allows for DNACPR decisions to be discussed comfortably.

Respondents identified that wider societal discussion of end-of-life issues such as advance care planning facilitated discussion of DNACPR and led to patients being better informed about the limitations of CPR and the meaning of a DNACPR decision. The suggestion that changes in political attitude could influence public perception of DNACPR orders was highlighted. In Japan, a country with a ‘*rapidly progressing*’ ageing population, one respondent said that ‘*the government is trying to advance the political policies of enlightening and spreading end-of-life care and advance directives*’*.* One respondent from Switzerland said that the national ‘*…liberal policy on assisted suicide*’ had enabled ‘*discussion of the achievements of palliative care and acknowledgment of its engagement.*’

#### Religion

Respondents from countries with strong religious majorities cited religion as having an impact on how patients and their families perceived DNACPR decisions. The respondent from Israel stated:

‘…religious orthodoxy has a very strong influence on reluctance to withhold resuscitation in Israel, and it is becoming more prominent.’

Respondents felt that religious beliefs could lead to misconceptions about CPR. A respondent from Brazil talked about Evangelical Christian teaching leading to a perception that treatment may have ‘*miracle possibilities*’. The respondent from Poland wrote that while the ‘*Roman Catholic Church is against futile therapy it is a common opinion that medical treatment (including CPR) should be instituted always.*’

A number of respondents stated that DNACPR decisions were often misinterpreted by patients or their families as a form of euthanasia. The respondent from Pakistan wrote:

‘… I have experienced initial resistance with regards to religious beliefs…people may associate a DNR order with euthanasia and it is important to clarify this… being a Muslim country there are very strong anti-euthanasia views here.’

While religion appeared generally to be presented by respondents as negatively influencing perceptions of DNACPR decisions, the respondent from Iceland saw the role of the church as positive in supporting the patient's right to refuse futile treatment.

#### Strength of individual autonomy

There was a distinction between those societies which valued individual autonomy and those where there was a more paternalistic attitude. The respondent from Singapore stated ‘*…in a country which is predominantly Chinese…patient autonomy is not the prevailing model for making end-of-life decisions.*’ A respondent from Spain described a ‘*culture…which support(s) a ‘paternalistic’ approach to the patient*’. Conversely, the respondent from Belgium felt that too much emphasis could be placed on respect for patient autonomy at the expense of other values such as ‘*trust*’ or ‘*care*’.

Responses suggest that societies in which the patient's family have a greater involvement in their care tend to give less value to individual patient autonomy: doctors would respect families' wishes to keep information from patients, rather than giving primacy to the patient's need to know.

Across cultures, making a DNACPR decision could be perceived by family as them ‘*giving up*’ on a loved one.

#### Economic factors

Unsurprisingly the structure of the health system and the resources available affected CPR decision-making. In Brazil, a publically-funded health system was felt to lead to patients lacking confidence in the financial power of the system and being suspicious of any decisions to limit treatment. In contrast the respondent from Norway described having a publically-funded health system as positive: ‘*The fact that health care in this country is free to every resident, and that we as physicians have no financial or other incentive to recommend one course of action vs another, contributes to trust.*’

Where healthcare was well resourced, respondents said that aggressive life-prolonging treatment was more likely to be expected. One respondent from Canada described a ‘*sense of entitlement of healthcare*’. In contrast ‘*severe resource limitation’* could make it ‘*difficult to motivate for resuscitation*’ (South Africa). As the respondent from Uganda wrote: ‘*We rarely attempt resuscitation…because we don’t have ITU etc.…I think most healthcare professionals here wouldn’t see the need for these (DNACPR) orders.*’

#### Medical culture

The influence of the culture of medicine on decision-making was highlighted by a number of respondents. They described a culture in which DNACPR decisions were seen as ‘*medical decisions to make and not shared decisions with patients*’ (New Zealand). Doctors were described as reluctant to consider the use of DNACPR: for some it was seen that prolongation of life was their primary goal, particularly in intensive care units, even when this may not be the ‘*primary wish of the patient*’ (Germany).

Many highlighted the importance of medical education in determining how DNACPRs were perceived and utilised: a lack of teaching around end-of-life care at medical school resulted in doctors that neither appreciated the value of a DNACPR decision nor had developed the communication skills necessary to discuss these decisions with patients and their families. Several respondents identified the need for teaching around end-of-life issues in medical school and in particular the teaching of medical ethics and law to student doctors.

## Discussion

Survey responses were received from geographically, culturally and economically diverse nations; almost all said that CPR is not always an appropriate intervention. The heterogeneity of responses highlights the complexities of decision-making surrounding resuscitation. The application of ethical principles to guide decision-making, in particular the value placed upon individual autonomy, are influenced by cultural context.

### Influences on DNACPR decision-making

#### Cultural

Societies in which the patient's family has great involvement in their care tend to give less value to the autonomy of the individual patient in determining their treatment; families often tend towards attempts to preserve life,^11^ but cultural attitudes towards death play a large role in determining how DNACPRs are perceived within a population.

Some authors have concluded that patients and families from religious communities are more likely to desire aggressive therapy when near death.^12^, ^13^ In contrast some argue that the Abrahamic religions do not support administering futile therapy.^14^ Our responses suggest that where religion has been cited as influencing negative perceptions of DNACPR, improving public awareness of the limitations of the procedure could lead to an improved perception of DNACPR decisions.

#### Economic

Increased treatment capabilities of modern medicine have heightened the public's expectations of what healthcare can currently achieve. This viewpoint was expressed in responses from both developed and developing nations. Patients often hold overoptimistic views about the limitations and consequences of CPR because of media portrayals.^15^

#### Politico-legal

One commonly identified obstacle to DNACPR implementation is ambiguity about how national law views decisions to withdraw or withhold treatment. In Taiwan, implementation of a national ‘Do Not Resuscitate’ policy is believed to have contributed to a significant reduction in the number of CPR attempts made in hospital.^16^ In the UK, the procedures for recording DNACPR decisions have changed as national guidelines were established^17^; what started as a ‘code’ buried in the medical records^18^ has become a bold form at the front of the notes for ease of recognition in an emergency.^19^

Where national policy and guidance exists, such in the UK, clinicians have a responsibility to be familiar with this. In absence of national policy, guidance has been provided by international organisations which should again direct clinicians decision-making.^20^

There is correlation between the perception of DNACPR orders and other aspects of end-of-life care, such as advance directives. The example of Switzerland demonstrates how public opinion towards end-of-life care can be influenced by changes in political attitude. Our responses from the Netherlands echo previously published views^21^ that the legalisation of euthanasia in the country has resulted in more open discussions about end-of-life issues.

#### The culture of medicine and the role of medical education

Medical education was highlighted as an important factor in determining how DNACPR orders were perceived and utilised.

Prior education about DNACPR orders significantly affects medical students' attitudes towards DNACPR^22^, ^23^, ^24^ and palliative care training makes doctors more likely to discuss limiting treatment options with patients.^25^, ^26^

Many respondents said that end-of-life care was now beginning to receive a greater emphasis in medical training; this is reflected in the literature.^27^, ^28^ Whether this will translate into better understanding and improved practice around resuscitation discussions and decisions remains to be seen.

#### Place of death

Reliable data on place of death globally does not currently exist, with only a small number of countries systemically collecting this data.^29^, ^30^, ^31^ However, the reports from respondents in this survey resonate with findings from previous research.^32^ Economic wealth and the culture within a country appear to be the biggest determinants of place of death. The most important social factor is the strength of extended family structures. This, coupled with limited access to intensive treatment, means that individuals are more likely to die at home.^6^ The biggest predictor of place of death is the availability of acute care.^33^ The UK, with a free national health service has one of the highest numbers of patients dying in hospital. Even in cultures where a strong family ‘duty to care’ for dying relatives exists, such as Taiwan, numbers of deaths within hospital are higher in urban areas where acute care is more easily accessed.^34^ Nevertheless, the Netherlands shows that even when acute care is readily accessible, it is still possible for the majority of patients to pass away outside of the hospital.^35^

Place of death is linked to the availability and use of palliative care and hospice services.^36^ In countries where these services are better established, there has been a recognition that patients should be supported to die at home if this is their wish.^35^ In Japan, despite a commitment to the development of palliative care and hospice services, there is sometimes a reluctance to use them as this may be felt to be an abandonment of a familial responsibility for a relative.^37^

#### Discussing DNACPR

Almost all of our respondents said that they routinely held discussions about DNACPR orders with their patient or the patient's family. However many respondents suggested that these conversations are, to varying extents, ‘*superficial*’ and most often result in patients doing what their doctors have recommended to them.

Although discussing CPR at all represents a huge cultural shift,^11^, ^38^ there is still scope for significant improvement: we should be evaluating ways to engage with individuals to think about what treatments they might want before they become ill. Contextualising CPR decisions within overall goals of care has been helpful in achieving this in both the US^39^ and the UK.^19^

### Limitations of the study

By searching for physicians who had previously published material on end-of-life care, only people who held a *specific* interest in this field were surveyed. Although some respondents intimated that some of their national colleagues were less appreciative of the utility of a DNACPR order, responses are still likely to reflect ‘best’ practice that occurs within the country. This may be compounded by respondents reporting different practice than that which actually occurs; DNACPR use in Italy and Spain, for example, is likely to be more widespread than international questionnaires suggest.^40^ The response rate of 44% introduces further potential bias, as does the fact that 22% of responders did not complete every question.

The greatest number of respondents from one particular country was six from the USA. For the majority of countries sampled, only one or two respondents were obtained. The responses are therefore not representative of national perceptions and use of DNACPR decisions. Heterogenic responses from Brazil and India highlighted this concern; the large geographical span and the great disparity in healthcare accessibility between the richest and poorest inhabitants of these two nations may have contributed. There were also regional disparities in DNACPR implementation in developed countries. A full international evaluation would require sampling from multiple clinicians from different healthcare settings, and translation of all available national policy guidance and documents. We believe that this would be a worthwhile endeavour, to help us learn from each other, and to understand more about the shifting populations which we all live among.

## Conclusion

This was an exploratory study demonstrating the heterogeneity of approaches to CPR decisions and the influence of societal and cultural factors. The challenges clinicians face when making CPR decisions are universal. Medical education must equip doctors for these decisions and discussions; policy makers can positively influence the debate.

## Conflict of interest statement

The authors have no conflicts of interest. The authors alone are responsible for the content and writing of the paper.

## Figures and Tables

**Table 1:** Influences on decision-making regarding Do Not Attempt Cardiopulmonary Resuscitation.

Societal/cultural	
Attitudes to death	“Culturally Indians are conscious of death to be “good” i.e., peaceful, spiritually meaningful, timely and with family in attendance, but are not able to reconcile this with curative possibilities offered by modern Medicine. Culturally the average Indian is unable to relate to a “probabilistic” model of prognostication and is used to an “emotional” assessment of situations.” (India) “Generally we don't talk about death directly to the patient. We generally think talking about death comes as bad luck.” (Japan) and similar in Singapore and Taiwan “Most patients and families are resistant to discuss it till late stages of disease because of fear of death and admission of futility…” (Lebanon) “American “can-do” attitude may be a barrier. A cultural aversion to death exists.” (USA) “…Death is considered sacred and so DNR orders are culturally prohibited by certain sections of people.” (Uganda) “Average life expectancy in SA is 50 years. Most patients are accepting (of death).” (South Africa)

Societal attitudes to assisted dying	“Forgoing CPR is often misinterpreted as a euthanasia. Legal regulations concerning foregoing CPR are not clear and many doctors decide for CPR in order to avoid legal consequences.” (Poland) “Sometimes I have experienced initial resistance with regards to religious beliefs regarding DNR orders. People may associate a DNR order with euthanasia and it is important to clarify this to them… being a Muslim country there are very strong anti euthanasia views here.” (Pakistan) “We practically do not have any euthanasia debate at all, probably because of good legal/medicolegal possibilities to provide efficient palliative care and also stop/or not initiate life sustaining care if indicated” (Sweden)

Wider societal discussion around end of life decisions	“Doctors seldom ask patients about advance directives or advance care planning.” (Japan) “There aren't any discussions connected with (Advance Directives)” (Poland) “More (DNACPR) discussions are being held as Singapore is rolling out advanced care planning*…*” (Singapore) “…discussions on DNR orders were deepened when discussions on Advance Directives came public” (Brazil) “Clear correlation in national debates about end-of-life” (France)

Role of religion	“Some religious factors, especially regarding some Evangelical Protestants patients/families who insist in the miracle possibility, often bring some difficult discussions. However, I see that these scenarios can be more easily dealt when professionals learn empathic communication skills.” (Brazil) “Religious teaching or perception that (DNACPRs are) unacceptable” (USA) “I believe that religious orthodoxy has a very strong influence on reluctance to withhold resuscitation in Israel, and it is becoming more prominent.” (Israel) “Poland is a catholic country. Although Roman Catholic Church is against futile therapy it is a common opinion that medical treatment (including CPR) should be always instituted.” (Poland) “Rights of patients to say no to treatment they don't think will help them, and it's part of our legislation. Churches have supported that view.” (Iceland)

Societies which value autonomy versus those with more paternalistic culture	“Being in a country which is predominantly Chinese (and Asian), patient autonomy is not the prevailing model for making end-of-life decisions. Rather decision-making is made collectively with the family and this raises problems when families want to hide certain information from the patient, example cancer diagnosis – collusion.” (Singapore) “Mediterranean cultures and cultural patterns support a ‘paternalistic’ approach to the patient (by family)”(Spain) “(Patients) always follow our recommendation… many people believe that doctors know best” (Saudi Arabia) “Great value attached to AUTONOMY of the patient, in my personal view, values as ‘trust’, ‘care’ are sometimes forgotten.” (Belgium) “Rights of patients to say no to treatment they don't think will help them, and it's part of our legislation” (Iceland)

Role of the family	“Many Korean family caregivers think that agreement on DNR decision is “not doing their best” for their patients. Therefore, they frequently ask the doctors to perform the most aggressive medical care, until the end-of-life. They frequently think that such attitude is righteous as a family.” (Korea) “Families in Pakistan are mostly very close knit and that too can sometimes result in resistance to DNR orders as often people feel they may be giving up on a loved one by agreeing to a DNR order.” (Pakistan)

Economic	
Structure of healthcare system	“The public hospitals assist patients in a very vulnerable situation, from the social-economic point of view, and they usually show preferences to a more paternalistic decision making process.” (Brazil) “Patients/families who had difficulties to ingress in the system (which is overloaded and often cannot accomplish the demand), more often feel distrust in the system, and thus, have greater difficulty to accept the possibility of end of life and limitations regarding treatments/life supports” (Brazil) “The fact that health care in this country is free to every resident, and that we as physicians have no financial or other incentive to recommend one course of action vs another, contributes to trust.” (Norway)

Resources available for healthcare	“With the relatively accelerated economic growth in the last 2 decades or so there is greater availability of, and expectations from modern healthcare. The well off have tended to be too aggressive in their demands from the medical profession and may insist on “doing everything”. In a death defying attitude they may demand therapies that are to physicians irrational, excessive and inappropriate. This is particularly so in the private healthcare sector which constitutes 80% of tertiary healthcare.” (India) “Economic privileges have allowed us to push the envelope regarding high-cost interventions and advocating for high-risk patients, even when survival seems unlikely” (USA) “Severe resource limitation factor into decision making.” (South Africa) “We rarely attempt resuscitation… because we don't have ITU etc. and most patients who die in hospital here are assumed to be at a terminal stage of some severe sepsis/HIV etc./unknown cause/severe anaemia/respiratory problem. I think most healthcare professionals here wouldn't see the need for these orders” (Uganda)

Political	
Government policy influence over end of life decision-making	“Many health care decisions and laws made by the central Government, are highly influenced by the political sign of the government instead of being evidence-based or ethically discussed” (Spain) “Preparation for end-of-life is currently about to boom in Japanese elderly because the aging of Japanese population is rapidly progressing… the government is trying to advance the political policies of enlightening and spreading EOL care and Advanced Directives.” (Japan) “Guidelines provide good help for decision making and reduces eventual fear and concern over medico-legal issues for the decision making doctor. Facilitates communication with patient and family members and reduces the risk that the decision is a personal expression of the doctor in charge” (Sweden) “Discussion of the achievements of palliative care and acknowledgement of its engagement … were solicited by the … quite liberal policy on assisted suicide.” (Switzerland)

The role of legislation	“Unfortunately due to the lack of legislation around end-of-life management, many patients with end stage illnesses undergo futile procedures” (Greece)

Culture of medicine	
Medical paternalism	“I do think a culture still exists (I'm not sure how prevalent) amongst some doctors that these are “medical” decisions for us to make – and not shared decisions to be made with patients. Part of this might of course be avoidance behaviour (of difficult conversations because of lack of skills/training/knowledge and support, and fear of harming the patient by these conversations) but some of it is still I suspect deeply ingrained medical paternalism” (New Zealand) “Once patients are being admitted to the ICU, their treatment is subject to the culture of ICUs with the primary aim to prolong life and provide life support, which may not be the primary wish of the patient.” (Germany)

The role of medical education, particularly training in medical ethics	“I think (hope) that greater teaching of law and ethics to health professionals (certainly to medical students) is slowly changing perceptions around these matters, alongside the greater emphasis on patient-centred care from the Quality and Safety movement and other sources.” (New Zealand) “In 2005, 60% of doctors from the university hospital didn't know the term DNAR! Hopefully it has changed since that time because we intensified education in this area including implementation of this issue into our medical university programme. However there are still many polish doctors who perform CPR as routine in every patient who arrests in the hospital!” (Poland) “Great variations (in healthcare professionals' views on DNACPR) exists based on cultural and religious aspects and also lack of knowledge of how to deal with patients and families after the DNR order is finalized… There is great gap of knowledge in general about ethics in medical practice and end-of-life issues is not even in the curriculum for medical students.” (Saudi Arabia)

The role of the physician's medical specialty and hospital environment	“With increased specialization of medical/surgical fields, many physicians look at their role in terms of maintenance of chronic care, endoscopy, diagnostic planning etc. but the DNR issue is not their business*…* Apathy and isolated thinking from physicians are real problems” (Canada) “Once patients are being admitted to the ICU, their treatment is subject to the culture of ICUs with the primary aim to prolong life and provide life support, which may not be the primary wish of the patient.” (Germany) “Scarcely any people in my new surroundings are interested in end of life care… In my previous hospital there was a general interest in giving adequate quality of care to patients in all phases of illness” (Spain)
