# Supplementary material for: Themes and variations: An exploratory international investigation into resuscitation decision-making
Source: Resuscitation. 2016 Jun;103:75–81. doi: 10.1016/j.resuscitation.2016.01.020 (PMC4879149; doi:10.1016/j.resuscitation.2016.01.020)
Supplement: Supplementary file 1 [file mmc1.doc]

**Appendix A. Questionnaire**

**Acknowledgment**

On the proposed publication would you like:

a. Acknowledgment

b. Anonymity

**Nationality**

Please state the country in which you practise:

**Local Practice**

1. Does your place of work have a method for making decisions on ‘not to attempt resuscitation’ for certain patients?

a. Yes (Please briefly outline this method)

b. No (Please go to question 4)

2. How do you communicate these decisions to other doctors in your institution?

a. Verbally

b. Written in the notes

c. By completing a pre-printed document

d. Electronically

e. Another method (please outline this)

3. How often do you discuss decisions about resuscitation with patients and/or their family?

a. Never

b. Rarely

c. Around half of the time

d. Most of the time

e. Always

4. In what setting do most patients die within your country?

a. Hospital

b. Home

c. Hospice

d. Nursing home

e. Elsewhere

5. Does national guidance exist for making resuscitation decisions in your country?

a. Yes

b. No (please go on to Question 7)

6. Please name the organisation that issues this guidance and what that guidance is? (Please give the full name of the organisation if you are able to)

7. Do you think that there should be a national policy/guidance for making resuscitation decisions or not? Please give reasons for your answer.

**National Perceptions**

We would like to gain some insight into how resuscitation decisions are viewed in your country. Please can you tell us:

1) Your personal opinion on Do-Not-Resuscitate orders? Please explain how your clinical experiences have contributed to your opinion.

2) What do you believe the general opinion is on Do-Not-Resuscitate orders amongst:

a) other healthcare professionals?

b) your patients?

c) the families of your patients?

*Thank you for answering the above questions. We are also interested in your opinion on how other aspects of life within your country have influenced how decisions about resuscitation are made. If you have time, please consider the following questions:*

1) What factors (eg cultural, economic, political, religious, social) do you think have influenced how Do-Not-Resuscitate orders are used and perceived in your country?

2) How does national opinion on Do-Not-Resuscitate orders correlate with opinion on other issues related to end of life care, such as Advance Directives and euthanasia?
