# Supplementary material for: Themes and variations: An exploratory international investigation into resuscitation decision-making
Source: Resuscitation. 2016 Jun;103:75–81. doi: 10.1016/j.resuscitation.2016.01.020 (PMC4879149; doi:10.1016/j.resuscitation.2016.01.020)
Supplement: Supplementary file 2 [file mmc2.docx]

**Appendix B. Invitation email**

Dear _________,

We have been conducting research into the use of Do-Not-Resuscitate orders in the UK, and we are interested in understanding international practices regarding this.

We have identified authors from around the world who have published on resuscitation decisions and/or other closely related matters; we enjoyed your article: _______ and were hoping that you might be able to assist us by completing our short questionnaire regarding Do-Not-Resuscitate practice in your country.

This questionnaire is attached as a word document and can also be found below within the text of this email.

Using the format you prefer, please could you complete as many questions as you have time to: we would rather receive some information than none at all. If you would prefer to receive the questions in another language, or would like to answer the questions in another language, please let us know.

We intend to publish collated responses, and would be happy to acknowledge your contribution. Alternatively, we are able to preserve your anonymity (stating only that we had an input from your country) should you so wish.

Please let us know if you have any questions for us.

With best wishes,

**Dr. Zoë Fritz** (Consultant Physician, Acute Medicine and Wellcome Fellow in Bioethics, Cambridge University Hospitals)

**Dr. Jonathan Fuld** (Consultant Physician, Acute and Respiratory Medicine, Cambridge University Hospitals)

**Alexandra Malyon** (Nurse Researcher, Cambridge University Hospitals)

**Alexander Gibbs** (4th Year Medical Student, Gonville and Caius College, University of Cambridge)

Department of Acute Medicine, Box 148

Cambridge University Hospitals, Hills Road, Cambridge,

CB2 0QQ

UK

Telephone: +44 1223 274597

Fax: +44 1223 348319
