# Supplementary material for: Themes and variations: An exploratory international investigation into resuscitation decision-making
Source: Resuscitation. 2016 Jun;103:75–81. doi: 10.1016/j.resuscitation.2016.01.020 (PMC4879149; doi:10.1016/j.resuscitation.2016.01.020)
Supplement: Supplementary file 3 [file mmc3.docx]

**Appendix C. Answers to questions 1-8**

| **Country of practice** | Does your place of work have a method for making decisions on ‘not to attempt resuscitation’ for certain patients? | How do you communicate these decisions to other doctors in your institution? | How often do you discuss decisions about resuscitation with patients and/or their family? | In what setting do most patients die within your country? | Does national guidance exist for making resuscitation decisions in your country? | Do you think that there should be a national policy/guidance for making resuscitation decisions or not? |
| --- | --- | --- | --- | --- | --- | --- |
| Argentina | Yes | Verbally | Rarely | Hospital | Yes | Yes |
| Australia 1 | Yes | Verbally, Written in the notes, By completing a pre-printed document | Around half of the time | Hospital | No | Yes |
| Australia 2 | Yes | Written in the notes | Most of the time | Hospital | Yes | Yes |
| Australia 3 | Yes | Verbally, Written in the notes, By completing a pre-printed document | Most of the time | Hospital | Yes | Yes |
| Austria | Yes | Written in the notes | Around half of the time | Hospital | No | Yes |
| Barbados | Yes | Verbally, Written in the notes | Around half of the time | Hospital | No | Yes |
| Belgium | Yes | Written in the notes, Electronically | Rarely, Around half of the time | Hospital | Yes | Yes |
| Brazil | Yes | Verbally, Written in the notes | Most of the time | Hospital, Home | Yes | Yes |
| Brunei | No | Verbally, Written in the notes | Rarely | Hospital | No | Yes |
| Canada 1 | Yes | Verbally, Written in the notes | Always | Hospital | No | - |
| Canada 2 | Yes | Verbally, Written in the notes, Electronically | Always | Hospital | Yes | Yes |
| Canada 3 | Yes | Written in the notes, By completing a pre-printed document | Most of the time | Hospital | Yes | Yes |
| Colombia | Yes | Verbally | Around half of the time | Hospital | No | Yes |
| Cuba | Yes | Verbally | Always | Home | - | - |
| Denmark | Yes | Electronically | Most of the time | Hospital | Yes | Yes |
| France 1 | Yes | By completing a pre-printed document | Most of the time | Hospital | Yes | Yes |
| France 2 | No | N/A | N/A | Hospital | Yes | - |
| Germany | Yes | Verbally, Written in the notes, Electronically | Always | Hospital | Yes | Yes |
| Greece | No | N/A | N/A | Hospital | No | Yes |
| Holland | Yes | Electronically | Around half of the time | Home | No | Yes |
| Holland 2 | Yes | Electronically | Around half of the time | Home | Yes | - |
| Holland 3 | Yes | Electronically | - | Hospital, Home | Yes | Yes |
| Hong Kong | Yes | Verbally, Written in the notes, By completing a pre-printed document | Around half of the time | Hospital | Yes | Yes |
| Hong Kong 2 | Yes | Written in the notes, By completing a pre-printed document | Always | Hospital | No | Yes |
| Hungary | Yes | Verbally | Rarely | Hospital | Yes | Yes |
| Iceland | Yes | Written in the notes, Electronically | Rarely | Hospital | No | Yes |
| India 1 | Yes | Verbally, Written in the notes | Always | Hospital, Home | No | Yes |
| India 2 | Yes | Verbally | Always | Home | No | - |
| India 3 | Yes | Written in the notes | Always | Home | No | Yes |
| Ireland | Yes | Written in the notes | Most of the time | Hospital | Yes | Yes |
| Ireland 2 | Yes | Written in the notes | Rarely | Hospital | Yes | Yes |
| Israel 1 | Yes | Verbally, Written in the notes | Around half of the time | Hospital | No | Yes |
| Israel 2 | Yes | Verbally, Written in the notes | Most of the time | Hospital | No | Yes |
| Israel 3 | Yes | Written in the notes | - | - | - | - |
| Italy | No | N/A | N/A | Hospital | Yes | - |
| Japan 1 | No | N/A | N/A | Hospital | No | Yes |
| Japan 2 | Yes | Written in the notes | Around half of the time | Hospital | No | Yes |
| Japan 3 | Yes | Verbally | Most of the time | Hospital | No | No |
| Lebanon | Yes | Verbally, Written in the notes, Other method | Most of the time | Hospital | Yes | Yes |
| Malaysia | No | N/A | N/A | Home | No | Yes |
| Malaysia 2 | Yes | Written in the notes | Rarely | Hospital | No | Yes |
| Malta | Yes | Written in the notes | Most of the time | Hospital, Home, Nursing home | No | Yes |
| New Zealand | Yes | By completing a pre-printed document | Always | Hospital | No | Yes |
| Norway | Yes | Verbally | Always | Hospital | Yes | Yes |
| Norway 2 | Yes | Written in the notes, Electronically | Rarely | Hospital | Yes | Yes |
| Pakistan | Yes | Written in the notes, Electronically | Always | Hospital | No | Yes |
| Poland | Yes | Written in the notes, By completing a pre-printed document | Most of the time | Hospital | No | Yes |
| Poland 2 | Yes | Verbally, Written in the notes | Always | Hospital, Home | No | Yes |
| Puerto Rico | Yes | By completing a pre-printed document | Always | Hospital | Yes | - |
| Saudi Arabia 1 | Yes | Written in the notes, Electronically | Always | Hospital | No | Yes |
| Saudi Arabia 2 | Yes | By completing a pre-printed document | Always | Hospital | Yes | Yes |
| Saudi Arabia 3 | Yes | Verbally | Most of the time | Hospital | No | Yes |
| Singapore 1 | Yes | By completing a pre-printed document | Most of the time | Hospital | No | Yes |
| Singapore 2 | Yes | Verbally, Written in the notes, By completing a pre-printed document | Most of the time | Hospital | No | Yes |
| Singapore 3 | Yes | Verbally, Written in the notes, Electronically | Most of the time | Home | No | No |
| Singapore 4 | Yes | By completing a pre-printed document | Always | Hospital | No | Yes |
| Singapore 5 | Yes | Verbally, By completing a pre-printed document | Around half of the time | Hospital | No | Yes |
| South Africa | No | N/A | N/A | Hospital | No | No |
| South Africa 2 | No | Verbally, Written in the notes | Rarely | Hospital | No | Yes |
| South Korea | Yes | By completing a pre-printed document | Always | Hospital | Yes | Yes |
| Spain 1 | Yes | By completing a pre-printed document | Always | Hospital | No | Yes |
| Spain 2 | Yes | Verbally, Written in the notes, By completing a pre-printed document | Most of the time | Hospital | Yes | No |
| Spain 3 | Yes | Verbally, Written in the notes, Electronically | Most of the time | Hospital | No | Yes |
| Sri Lanka | Yes | Written in the notes | Most of the time | Hospital | Yes | - |
| Sweden | Yes | Verbally, Written in the notes, By completing a pre-printed document, Electronically | Most of the time | - | Yes | Yes |
| Switzerland 1 | Yes | Verbally, Written in the notes, By completing a pre-printed document | Most of the time | Hospital | Yes | Yes |
| Switzerland 2 | Yes | Verbally, Written in the notes, Electronically | Around half of the time | - | Yes | Yes |
| Taiwan 1 | Yes | Written in the notes, By completing a pre-printed document, Other method | Around half of the time | Hospital | Yes | - |
| Taiwan 2 | Yes | Verbally | Rarely | Hospital | Yes | Yes |
| UAE | Yes | Verbally, Written in the notes | Around half of the time | Hospital | No | Yes |
| Uganda | No | N/A | N/A | Home | No | Yes |
| Uganda 2 | Yes | Written in the notes | Always | Home | No | Yes |
| USA 1 | Yes | Verbally, Written in the notes, By completing a pre-printed document, Electronically | Always | - | No | - |
| USA 2 | Yes | Written in the notes | Always | Home | No | - |
| USA 3 | - | - | - | Hospital | Yes | Yes |
| USA 4 | Yes | Verbally, Electronically | Always | Hospital | Yes | Yes |
| USA 5 | Yes | Electronically | Most of the time | Hospital | No | Yes |
| USA 6 | Yes | Verbally, Written in the notes, Electronically | Most of the time | - | No | Yes |
